# Lactate Biosensing for Reliable On-Body Sweat Analysis

**DOI:** 10.1021/acssensors.1c01009

**Published:** 2021-07-06

**Authors:** Xing Xuan, Clara Pérez-Ràfols, Chen Chen, Maria Cuartero, Gaston A. Crespo

**Affiliations:** Department of Chemistry, School of Engineering Sciences in Chemistry, Biotechnology and Health, KTH Royal Institute of Technology, Teknikringen 30, SE-100 44 Stockholm, Sweden

**Keywords:** lactate biosensor, diffusion limiting membranes, real-time monitoring, sweat analysis, wearable sensors

## Abstract

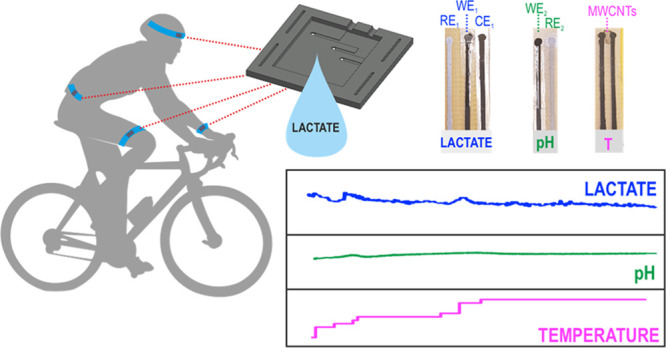

Wearable lactate sensors for sweat analysis are highly appealing for both the sports and healthcare fields. Electrochemical biosensing is the approach most widely used for lactate determination, and this technology generally demonstrates a linear range of response far below the expected lactate levels in sweat together with a high influence of pH and temperature. In this work, we present a novel analytical strategy based on the restriction of the lactate flux that reaches the enzyme lactate oxidase, which is immobilized in the biosensor core. This is accomplished by means of an outer plasticized polymeric layer containing the quaternary salt tetradodecylammonium tetrakis(4-chlorophenyl) borate (traditionally known as ETH500). Also, this layer prevents the enzyme from being in direct contact with the sample, and hence, any influence with the pH and temperature is dramatically reduced. An expanded limit of detection in the millimolar range (from 1 to 50 mM) is demonstrated with this new biosensor, in addition to an acceptable response time; appropriate repeatability, reproducibility, and reversibility (variations lower than 5% for the sensitivity); good resiliency; excellent selectivity; low drift; negligible influence of the flow rate; and extraordinary correlation (Pearson coefficient of 0.97) with a standardized method for lactate detection such as ion chromatography (through analysis of 22 sweat samples collected from 6 different subjects performing cycling or running). The developed lactate biosensor is suitable for on-body sweat lactate monitoring via a microfluidic epidermal patch additionally containing pH and temperature sensors. This applicability was demonstrated in three different body locations (forehead, thigh, and back) in a total of five on-body tests while cycling, achieving appropriate performance and validation. Moreover, the epidermal patch for lactate sensing is convenient for the analysis of sweat stimulated by iontophoresis in the subjects’ arm, which is of great potential toward healthcare applications.

Wearable chemical sensing for real-time and continuous monitoring of sweat composition is a very attractive concept for sport performance, as physiological information can be acquired without disturbing athletes during exercise.^1^ In particular, lactate is considered an important biomarker for such purpose due to its involvement in anaerobic metabolism.^2^ In a strong physical activity, undesired local accumulation of lactate may occur in the working muscle, this being manifested in the form of soreness, pain, and fatigue.^3^ Thus, changes in the concentration of lactate in blood are frequently used to monitor endurance sport in elite athletes through the well-known threshold curve.^2^ On the other hand, lactate determination in sweat is not as common yet, but some authors have pointed out a possible correlation between sweat lactate and exercise intensity.^4,5^ Some particular studies have reported on the need of monitoring sweat close to the active muscle to find such correlations.^5^ In addition, lactate in sweat is believed to provide unique information about the general health status of the individual, including pressure ischemia and insufficient oxidative metabolism.^6^ As a result, the real-time and continuous monitoring of lactate in sweat has been claimed as a rich source of information to preserve the health status of athletes while maximizing their sport performance.^5,7^

Electrochemical sensors are particularly convenient in the wearable sensing technology owing to their simplicity, high versatility, low cost, and great capacity for implementation into wearable devices.^8,9^ In the specific case of lactate detection in sweat, several nonenzymatic^10−12^ and enzymatic^13−16^ sensors have been reported in the last years, with electrochemical enzyme-based biosensors being the basis of the majority of commercial portable lactate meters for blood tests. Interestingly, these devices have been also used for the detection of lactate in sweat,^17^ although their accuracy strongly depends on the sweat collection method. This is known to traditionally suffer from issues related to uncontrolled sample evaporation.^18^ Consequently, the implementation of lactate biosensors in a wearable configuration, thus avoiding sample collection, is essential for its success.

In such direction, the most commonly used concept is lactate oxidase (LOx) as the enzyme^19,20^ in first-generation biosensors that use Prussian blue (PB) to mediate hydrogen peroxide (subproduct of the enzymatic reaction) detection at mild applied potentials.^13,16^ For example, Cheng and co-workers employed this approach to develop a textile wearable sensor based on gold fiber electrodes that presented a linear response up to 5 mM,^13^ which is not enough to cover the expected ranges in sweat while doing sport (lactate levels expected are up to ca. 25 mM or even higher). Vinoth et al. reported on a wearable device for the simultaneous determination of sodium, potassium, pH, and lactate with a linear range of response (LRR) from 1 to 25 mM for the latter, but with the sensitivity being highly affected by pH and temperature.^16^ Nevertheless, on-body measurements during cycling resulted in lower values than those measured by high-performance liquid chromatography, which deserves attention toward reliable measurements. In a similar direction, Gao et al. reported a wearable sensor array for the simultaneous determination of lactate, glucose, sodium, potassium, and temperature.^21^ This lactate biosensor showed a relatively wide LRR for lactate (2–30 mM), and the authors claimed that it did not require calibration prior to on-body implementation. However, data for the validation of on-body measurements against a reference analytical technique were only shown for glucose and sodium, with no tangible information regarding the reliability of on-body lactate measurements. Indeed, reliability in on-body determination of lactate is an issue that is not usually properly addressed in the literature.^12,21,22^

Lactate biosensing has also been demonstrated with dehydrogenase (LDH) enzyme,^23^ although the need of the nicotinamide adenine dinucleotide (NAD^+^) cofactor and proton coupling make it difficult to realize this enzyme as the core of an effective wearable sensor. Nevertheless, LDH biosensors are very attractive for other kinds of applications, such as extracellular measurements. Other options for lactate biosensing have explored second-generation biosensors using tetrathiafulvalene (TTF) as the redox mediator.^14,15^ The group of Wang presented a temporary tattoo biosensor with an LRR from 1 to 20 mM that was used in certain on-body tests with impressive results (nice correlation with the validation method).^14^ Payne et al. reported on a series of considerations about the effect of pH, temperature, and other ions in the amperometric response of TTF-LOx biosensors before being adapted for on-body measurements.^15^ Alternatively, the group of Karyakin used enzyme engineering to increase the apparent Michaelis constant of LOx and hence extend the LRR (up to 500 mM).^24^ Despite the on-body use of these sensors being not totally demonstrated, these works highlighted the importance of further investigations on wearable lactate sensing technology going in the direction of reproducible and extended LRR that covers expected levels in sweat.

Thus, although some efforts have already been made toward real-time wearable sensing of lactate in sweat, most of the reported works fail to meet at least one of the following indispensable criteria: (i) adequate LRR that covers lactate levels in sweat; (ii) nondependence to other sweat parameters such as pH, temperature, or interfering compounds,; and (iii) demonstrated reliability of on-body measurements. In this direction, we present a new lactate biosensor with unprecedented LRR from 1 to 50 mM in sweat. Such an impressive performance is achieved by means of the incorporation of an outer layer to the PB-LOx electrode. The layer is composed of a polymer, plasticizer, and lipophilic salt to limit the flux of lactate that reaches the LOx enzyme. Advantageously, the outer layer does not affect the response time upon lactate concentration changes and indeed prevents the biosensor response from being affected by pH, temperature, and other ions in the sample. This sort of outer layers has been denoted as ″diffusion limiting membranes″ in the literature and have been widely used to avoid the direct contact of the enzyme-based electrode with the sample solution.^25^ Beyond the expected partition of the analyte between such outer membrane and sample phases, the film porosity has been reported to affect the LRR of the resulting biosensor, with lower porosities providing a more efficient restriction toward lactate diffusion, avoiding enzyme saturation, and somehow controlling the presence of interfering species.^25,26^ The new lactate biosensor has been successfully implemented in a wearable epidermal patch together with pH and temperature sensors, being suitable for lactate measurements encompassing both natural and stimulated perspiration, as herein demonstrated.

## Experimental Section

### Preparation of Lactate, T, and pH Sensors

**Figure 1:**
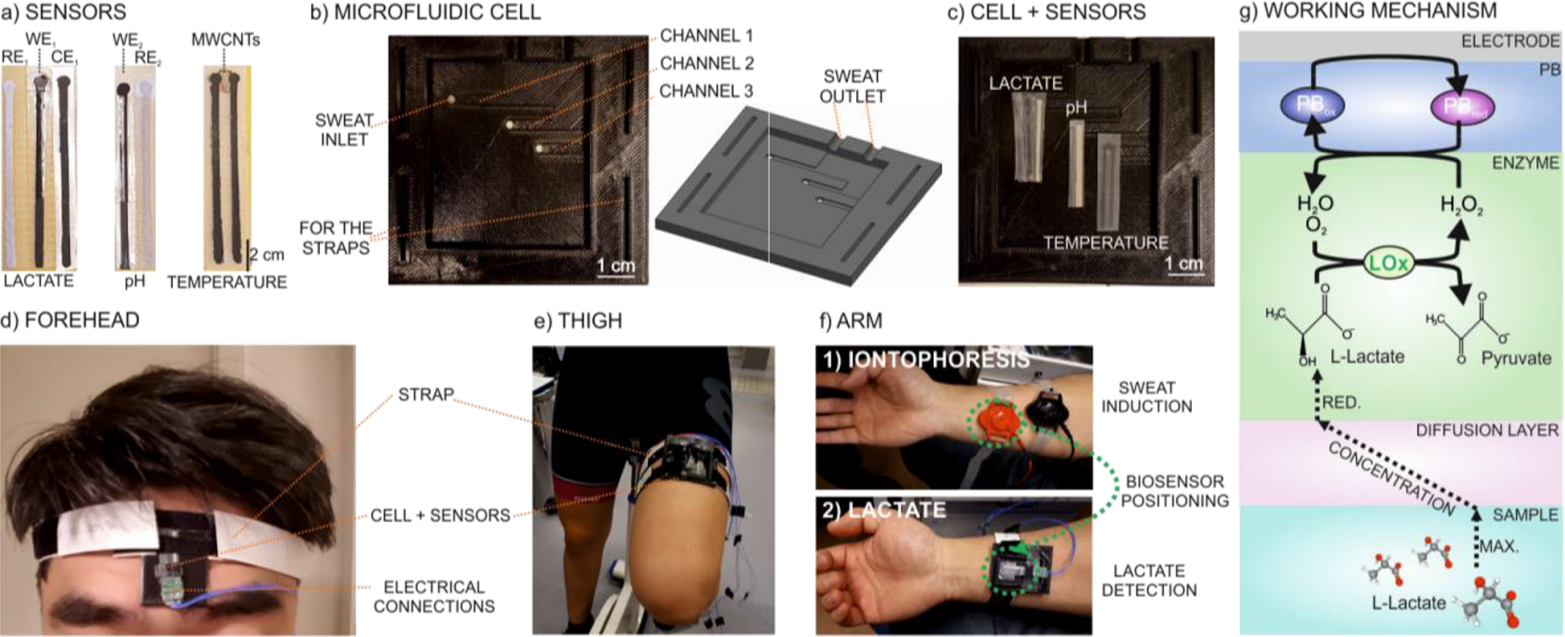
Images of (a) lactate, pH, and T sensors. (b) Epidermal patch with three microfluidic channels. (c) Epidermal patch with the sensors. Attachment of the epidermal patch in (d) the forehead and (e) thigh during cycling and (f) in the arm after iontophoresis. (g) Illustration of the sensing mechanism underlying lactate detection in sweat.

Lactate, pH, and temperature measurements were accomplished by amperometry (application of −50 mV), potentiometry, and resistance readouts, respectively. A total of seven electrodes were fabricated (Figure 1a): three for lactate (working, reference, and counter: WE_1_, RE_1_, and CE_1_), two for pH (working and reference: WE_2_ and RE_2_), and another two for temperature (two carbon electrodes). All electrodes were manually screen-printed using either carbon (working and counter electrodes as well as the temperature sensor) or Ag/AgCl (reference electrodes) ink. The paths consisted of a rectangular trace (3 × 17 × 0.1 mm) with a circle ending (diameter of 2 mm). The layer-by-layer structure of each electrode is shown in Figure S1 in the Supporting Information. Briefly, the lactate biosensor was based on three layers: Prussian blue (PB) as the redox mediator; lactate oxidase (LOx) as the enzyme; and a membrane containing tetradodecylammonium tetrakis(4-chlorophenyl) borate (ETH 500), polyvinyl chloride (PVC), and bis(2-ethylhexyl) sebacate (DOS). The pH sensor consisted of a polyaniline (PANI) electrode, and temperature measurements were based on the electrical connection of two carbon electrodes by multiwalled carbon nanotubes (MWCNTs).^27^ Details for electrode fabrication are provided in the Supporting Information.

### Fabrication of Epidermal Patches

The microfluidic cells, containing either three (Figure 1b) or only one microfluidic channel (Figure S2), were designed with the AutoCAD software and fabricated by 3D printing equipment. The cell was made of flexible polyurethane and consisted of inlets (1.5 mm diameter hole) implemented in the microfluidic channels (30 × 1.5 × 0.1 mm) to collect and flow the sweat during perspiration. The sensors in each channel were allocated at the same distance from the inlet to ensure simultaneity in the measurements. The presence of two outlets ensures that sweat accumulation at the end of the microfluidic channels is avoided. In the case of the cell containing the three channels, one of each is dedicated to lactate, pH, and T sensors (Figure 1c), whereas only the lactate biosensor is placed in the one-channel cell (Figure S2). To be attached to the skin, the part of the microfluidic cell opposite to that in where the sensors are implemented contains a double adhesive tape (medical grade). In addition, the patch is attached in the different parts of the body by means of straps. Figure 1d–f presents images of the epidermal patch positioned in the forehead, thigh, and arm, the latter after applying iontophoresis.

## Results and Discussion

### Investigation of Different PVC Membranes as Diffusion Limiting Layers in Lactate Biosensing

The sensing mechanism underlying the lactate detection developed in this paper is illustrated in Figure 1g. Essentially, the working principle is based on the conversion of lactate to pyruvate by enzymatic reaction with LOx, which results in the formation of hydrogen peroxide as the subproduct. This is then detected through PB as the redox mediator: upon activation at a constant potential, the PB layer in its original (oxidized) state (PB_ox_) is electrochemically reduced to PB_red_, which is then oxidized back to PB_ox_ in the presence of the hydrogen peroxide. The latter conversion occurs spontaneously and manifests in a more negative current at increasing concentration of peroxide and, hence, increasing concentration of lactate reaching the enzyme. The readout principle has been previously reported not only for the development of lactate sensors but also others, all relying on the formation of hydrogen peroxide through the enzymatic conversion of the analyte.^13,28^

**Figure 2:**
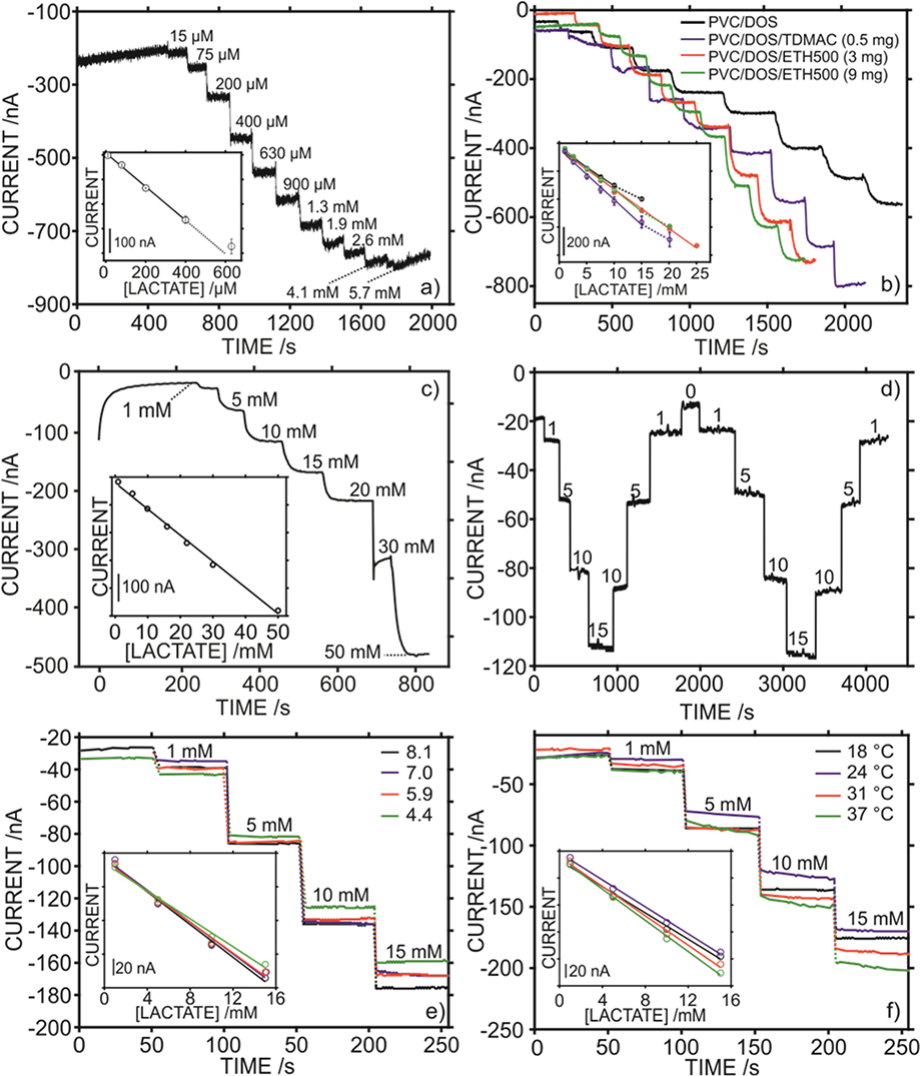
Dynamic responses and corresponding calibration curves (insets) carried out in phosphate buffer 0.1 M at increasing lactate concentrations using biosensors (a) without and (b) with an outer polymeric layer. (c) Chronoamperometric response of the optimized biosensor. (d) Dynamic response for reversibility studies. Dynamic amperometric response and corresponding calibration curves (insets) carried out (e) at room temperature and at different pH values and (f) at pH 8.1 and different temperatures.

Figure 2a displays the dynamic amperometric response at an applied potential of −50 mV at increasing lactate concentrations in 0.1 M phosphate buffer and using a biosensor based on a double-layer design: a PB film and the LOx enzyme immobilized in a Nafion matrix on top. As observed, the LRR is displayed from 15 to 400 μM, with a sensitivity of −641 ± 21 nA mM^–1^ (*n* = 3 electrodes), limit of detection (LD) of 32.6 μM (calculated as the lactate concentration corresponding to a signal-to-noise ratio of 3), and response time of <8 s (calculated as the time needed to reach the 95% of the steady-state signal) in the entire LRR. Then, the linear response deteriorates because of the enzyme saturation from 630 μM lactate concentration (inset in Figure 2a).

Despite this result being promising to be used as a calibration graph for the detection of unknown lactate concentrations in samples, the LRR is very low compared to the expected levels of lactate in any undiluted biological fluid: 3.7–50 mM in sweat, 0.5–1 mM in urine, 3–6 mM in plasma, 1–35 mM in interstitial fluid, 0.05–0.37 mM in saliva, and 1–5 mM in tears.^6,29−32^ Also, the response was slightly noisy, which is likely explained by an effect of the stirring of the sample solution over the lactate additions. Notably, other polymeric materials rather than Nafion have also been proposed in the literature to entrap LOx. This is the case for chitosan.^16,33−35^ However, these sensors also presented a reduced LRR.

It is therefore necessary to investigate how to develop a lactate biosensor presenting an LRR in the millimolar range rather than in the micromolar one together with a less noisy signal, minimizing any error in lactate quantification. Our proposal is the implementation of an outer layer on top of the enzyme aiming to provide a partition of the lactate available in the sample solution into that layer. Thus, the flux of lactate reaching the enzyme is reduced compared to that achieved with the bare enzyme directly immersed in the bulk solution (see Figure 1g). As a result, the saturation of the LOx is expected at a higher lactate concentration in the sample. This outer layer is traditionally called as ″diffusion limiting layer″ and has been proposed in the form of polymeric films with thicknesses ranging from 10 to 50 μm for other analytes.^26,36−38^ Specifically, we have evaluated PVC-based membranes deposited on top of the PB-LOx biosensor.

Figure 2b presents the dynamic responses and corresponding calibration graphs in 0.1 M phosphate buffer for lactate biosensors prepared with different outer layers: (i) a mixture of PVC and DOS [membrane M1], (ii) PVC–DOS matrix with 0.5 wt % tridodecylmethylammonium chloride (TDMAC) [membrane M2], and (iii) PVC–DOS matrix with 3 and 9 wt % of ETH 500 [membranes M3 and M4]. Note that DOS is a common plasticizer to create appropriate PVC-based membranes used in electrochemistry,^39^ TDMAC is an anion exchanger, and ETH 500 is a lipophilic electrolyte used to reduce the electrical resistance^40^ while increasing the hydrophobicity of the membrane.^41^ Importantly, recent cytotoxicity studies performed in our research group revealed that these compounds have no visible effect in a time frame from 0 to 96 h when incubated at different conditions with fibroblasts.^42^ In that study, even the leaching effect of the DOS plasticizer was discarded from potential toxicity. Notably, because the biosensor is integrated into the epidermal patch for the on-body tests, any cytotoxicity risk arisen from the components of the outer layer was minimized (or almost inexistent). For the detailed membrane compositions, the reader is referred to Table S1**.**

All the biosensors prepared with a PVC-based outer layer showed a shift of the LRR toward higher lactate concentrations (in the millimolar range) compared to the electrode prepared without any outer layer (Figure 2a *versus*Figure 2b). More specifically, the PVC–DOS membrane (M1) provided an LRR from 1 to 10 mM, with a sensitivity of −26.4 ± 0.7 nA mM^–1^ (*n* = 3 electrodes), LD of 0.10 mM, and response time of <86 s in the entire LRR. Then, the PVC–DOS–TDMAC membrane (M2) showed an LRR from 1 to 15 mM, with a sensitivity of −35 ± 2 nA mM^–1^ (*n* = 3 electrodes), LD of 0.13 mM, and response time of <79 s. Finally, the membranes with 3 and 9 wt % of ETH500 (M3 and M4) displayed LRRs of 1–25 and 1–15 mM, sensitivities of −29.3 ± 0.6 nA mM^–1^ (*n* = 3 electrodes) and −31.2 ± 0.9 nA mM^–1^ (*n* = 3 electrodes), LDs of 0.10 and 0.17 mM, and response times of <85 and <67 s, respectively. Overall, these experiments pointed out the right direction toward an appropriate LRR to detect lactate in undiluted biological fluids, confirming hence the core hypothesis of the working mechanism of the biosensor response based on a reduction of the flux of lactate reaching the enzyme layer (see Figure 1g).

The sensitivity of all the biosensors prepared with the outer PVC membrane was in the order of ca. 20 times lower than that displayed by the biosensor without any outer layer. This reduction in the sensitivity is in principle expected because the same enzymatic activity (i.e., the same PB-LOx configuration) is used in a wider lactate range in the case of biosensors fabricated with the polymeric outer membrane. Then, any difference in the LRR and/or the response time between the PVC-based biosensors is ascribed to a different partition of lactate in such membranes, as the thickness was kept to ca.10 μm (measured with profilometer measurements) in all the cases.

For the membrane containing the anion-exchanger TDMAC, it has been already demonstrated in the literature that quaternary ammonium salts (also in the form of ionic liquids) act as mobile carriers for lactate in plasticized PVC membranes.^43,44^ The permeation flux of lactate from a feed phase (analogous to the sample solution) to a receiving one (the enzyme layer in the case of our biosensor) through the membrane was unequivocally visualized. Interestingly, the net lactate flux from one phase to the other was independent of the amount of the quaternary ammonium salt in the plasticized PVC membrane. Indeed, we realized the same effect in our experiments, and practically the same calibration graph was obtained when the TDMAC was present in the range from 0.1 to 2.7 wt %. However, the response presented in Figure 2b for the biosensor prepared with the PVC/DOS/TDMAC outer layer was found to gradually deteriorate with subsequent calibrations in such a way that both the sensitivity and the lactate concentration corresponding to the enzyme saturation (i.e., shorter LRR) decreased. As a result, this biosensor was categorized as not convenient for further experiments.

Concerning the biosensors prepared with the PVC/DOS/ETH500 outer layer, previous studies have demonstrated that ETH 500 decreases water uptake and increases membrane lipophilicity, thereby reducing the resistance of plasticized PVC membranes.^40,45^ We observed that membranes containing 3 wt % ETH500 result in a wider LRR than those containing TDMAC or no additive and, more importantly and contrarily to membranes containing TDMAC, the response was reproducible not only between electrodes but also for consecutive calibrations. The sensor response was found to be dependent on the amount of ETH 500 in the plasticized polymeric membrane, with a higher content (i.e., 9 wt %) displaying a slightly narrower LRR. This deterioration in the sensor linearity is likely related to an overimproved permeation of lactate, which weakens the barrier effect of the membrane. Thus, the PVC/DOS/ETH500 membrane with a 3 wt % content of ETH500 was selected for further studies.

Finally, regarding the thickness of the outer polymeric layer, this was calculated to be close to 10 μm (by means of profilometer measurements) for deposited volumes ranging from 1.5 to 3 μL, which was an appropriate amount to ensure the full coverage of the working electrode surface without spreading the cocktail-drop to the entire chip and hence causing undesired short circuit. It was expected that the thickness of this layer modulates the analytical performance of the sensor: the thicker the layer is, the larger is the linear range, at the expense of both diminished sensitivity and longer response time. In this regard and after optimization, we found that 10 μm was a compromise for an operative situation ensuring the good performance of the sensor for sweat measurements.

### Evaluation of the Analytical Performances of the New Lactate Biosensor

The analytical performance of the developed lactate biosensor, as well as pH and T sensors, was first evaluated in batch mode and using artificial sweat as background. Figure 2c presents the dynamic response of the new biosensor at increasing lactate concentrations, with the inset showing the corresponding calibration graph. The lactate biosensor presented an LRR from 1 to 50 mM, with a sensitivity of −9.4 nA mM^–1^ (correlation coefficient of *R*^2^ = 0.997), intercept of −21.7 nA, LD of 0.11 mM, and response time of <55 s in the entire LRR. Response repeatability was calculated from three consecutive calibration curves using the same electrode (Figure S3a), obtaining a sensitivity of −8.5 ± 0.3 nA mM^–1^ and an intercept of −43.0 ± 1.5 nA, which correspond to %RSD of 4.1 and 3.4, respectively. Good reproducibility was also observed for the response provided by three twin sensors (sensitivity of −8.8 ± 0.3 nA mM^–1^ and intercept of −35 ± 8 nA, *n* = 3 different electrodes, Figure S3b).

The midterm drifts (Figure S4 in the Supporting Information) displayed by the biosensor at low (2 mM) and high (15 mM) lactate concentrations were 5.7 and 11.6 nA h^–1^, respectively, over a 1 h period. These are acceptable values that will represent an error in lactate concentration always lower than the 4% in case of medium-term continuous measurements. Then, the response of the lactate biosensor toward common compounds found in sweat was evaluated by adding concentrations of 0.25 mM glucose, 0.1 mM pyruvate, 0.1 mM ascorbic acid (AA), and 0.1 mM uric acid (UA) to a 5 mM lactate solution and registering any variation in the amperometric response (Figure S3c in the Supporting Information). An almost negligible response was observed for all these compounds, further demonstrating the ability of the developed lactate biosensor to directly measure sweat (i.e., no need for sample dilution or pretreatment). Resiliency tests were carried out by recording calibration curves before and after the application of a strong torsion strain to the biosensor (Figure S5 in the Supporting Information). Both slope and intercept were maintained after 30 torsions at 45°, with %RSD of 3 and 4%, respectively.

The potentiometric pH sensor was based on a conductive PANI film, as reported in previous works.^46^Figure S6a in the Supporting Information shows a calibration curve as the average of three electrodes over a pH range from 4.5 to 8.5, which covers normal pH values in sweat. A Nernstian response was observed, with a slope of 59.1 ± 0.6 mV and intercept of 387.1 ± 12 mV. Then, the temperature sensor utilized the chemoresistor properties of MWCNT: electrical resistance is dependent on the temperature. As observed in Figure S6b in the Supporting Information, the temperature sensor showed rather good performance in the temperature range from 20 to 40 °C, with a slope of 387.7 Ω/°C and intercept of 310.8 kΩ.

Aiming for on-body determination of lactate in sweat during exercise, the developed lactate biosensor was implemented in a custom-made (3D printed) epidermal patch, the design of which was shown in Figure 1c. The sample enters through the inlets, flows along each of the microfluidic channels (in which the sensors are allocated), and leaves the cell through the outlet. Conveniently, the analytical performance of the lactate biosensor after its implementation in the microfluidic cell was evaluated in flow mode by means of a peristaltic pump. As sweat rate is not constant during a training session and differs between individuals, any influence of the flow rate in the biosensor response was firstly evaluated. Flow rates between 0 and 12.5 μL min^–1^ were selected for this purpose. Taking into account that the area of the microfluidic channel is 0.9 cm^2^, such flow rates correspond to a sweat rate range from 0 to ca. 13.8 μL cm^–2^ min^–1^. This range covers the typical human sweat rate, which may range from 2 to 7 μL cm^–2^ min^–1^ depending on the subject and body part.^47^ Overall, we found a negligible influence of the flow rate in the amperometric response of the biosensor (Figure S7a,b in the Supporting Information). Thus, the steady-state signal reached for a constant concentration of lactate (5 mM) in artificial sweat did not show any significant variation when the flow rate was stopped or increased in steps of 2.5 μL min^–1^. Lactate additions before and after testing the influence of the flow rate additionally confirmed an adequate sensor performance.

Figure 2d presents the dynamic response observed in the evaluation of the reversibility of the biosensor amperometric response by means of decreasing and increasing the lactate concentration in an artificial sweat background. The biosensor exhibited an average calibration curve with a slope of −6.4 ± 0.3 nA mM^–1^ and an intercept of −19.9 ± 1.8 nA (Figure S7c), with RSD% values of 5 and 9%, respectively. Notably, the sensitivity was slightly lower than that observed in the batch mode studies (−6.4 *versus* −8.5 nA mM^–1^), which is in principle expected as a result of the change in the mass transport regime of lactate to the electrode.^48^Figure 2e shows the dynamic responses of the lactate biosensor at different pH values of the background solution (ranging from 4.4 to 8.1) with the corresponding calibration graphs (inset). Variation coefficients in the range of 7% for both the slope and intercept were observed. These variations are only slightly higher than those observed in reproducibility and reversibility studies and indeed much lower than pH variations reported in the literature for other lactate amperometric biosensors based on LOx.^49,50^

The outer PVC membrane in the biosensor tailoring is likely responsible for the absence of any pH influence in its response, as the transport of protons from the bulk solution to the enzyme layer is expected to be eliminated. This was confirmed by the almost negligible potentiometric response of the PVC/DOS/ETH500 membrane interrogated in potentiometric mode (Figure S8 in the Supporting Information). The resulting calibration curve at decreasing pH presented a slope of only 7 mV dec^–1^ compared to the theoretical Nernstian slope of 59.1 mV dec^–1^. Moreover, no significant changes in the biosensor response were found when the temperature was varied in the range from 18 to 37 °C (Figure 2f), with variation coefficients of 5 and 16% for the slope and intercept, respectively. Once more, these variations were lower in comparison with previous works reported in the literature.^27^

### Off-Site Validation of the Lactate Biosensor

Prior to usage in on-body measurements, the biosensor was validated by comparing the lactate concentration observed in 22 sweat samples with the results provided by IC as the gold standard technique. The samples were collected using the modified version of the regional absorbent pad recently published by our group.^51^ For this study, six different subjects were recruited and sweat samples were obtained from different body parts while either running or cycling over different time periods. Table 1 presents the conditions for the sample collection together with the results obtained with the epidermal patch and IC. Notably, lactate concentration in all the samples ranged between 9 and 20 mM, which is within the expected levels in sweat. Then, the differences between the results provided by the two techniques are in the range from ca. 2 to 11%, with most of the samples presenting less than 7% of difference. Furthermore, the correlation plot of the biosensor *versus* the IC results showed a linear regression with a slope of 0.90 ± 0.10, intercept of 1.1 ± 1.3, correlation coefficient (*R*^2^) of 0.95, and Pearson coefficient of 0.97 (Figure S9). These results pointed out an excellent correlation between both techniques.

**Table 1:** Lactate Concentration Observed in Sweat Samples by Means of the Epidermal Patch Containing the Lactate Biosensor Operating in Flow Mode and Ion Chromatography as the Gold Standard Technique^a^

				lactate (mM)		
subject	physical activity	collection time (min)	zone	IC	sensor	diff. (%)
1	cycling	30–40	forehead	14.6	15.5	6.2
2	running	20–30	back	9.6	10.1	5.2
		30–40	back	9.8	10.5	7.1
3	cycling	20–30	forehead	12.0	11.3	5.8
		30–40	forehead	11.7	11.3	3.4
4	cycling	10–20	back	12.5	13.7	9.6
		20–30	back	12.5	13.7	9.6
		30–40	back	13.2	12.5	5.3
		30–40	leg	18.6	19.5	4.8
5	cycling	10–20	forehead	10.7	10.9	1.9
		20–30	forehead	11.3	11.9	5.3
		30–40	forehead	10.2	11.4	11.7
		10–20	back	10.0	9.4	6.0
		20–30	back	10.4	10.9	4.8
		30–40	back	10.7	9.9	7.5
		30–40	leg	18.2	19.5	7.1
6	running	10–20	forehead	13.2	12.7	3.8
		20–30	forehead	13.7	14.2	3.6
		30–40	forehead	13.7	13.4	2.2
		40–50	forehead	14.9	14.2	4.7
		30–40	shoulder	18.2	18.9	3.8
		40–50	shoulder	17.9	18.2	1.7

a The differences between the results provided by the IC and the epidermal patch are provided in percentage.

### Analytical Application of the Developed Lactate Biosensor: On-Body Measurements of Sweat Lactate

The applicability of the developed epidermal patch containing the new lactate biosensor (and also pH and T sensors) for on-body measurements of sweat lactate was assessed in two different scenarios: (i) analysis of sweat naturally generated during cycling (toward sport performance monitoring) and (ii) analysis of sweat locally induced by means of iontophoresis (toward healthcare applications). In the first case, a total of five on-body tests (T1–T5) involving different subjects and body locations (forehead, back, and thigh) were accomplished. The enrolled subjects performed cycling activities based on ca. 75 min exercise program divided into 4 periods of 15 min each (warm up, low/middle effort level, middle/high effort level, and cool down) with 5 min rest between each stage.

**Figure 3:**
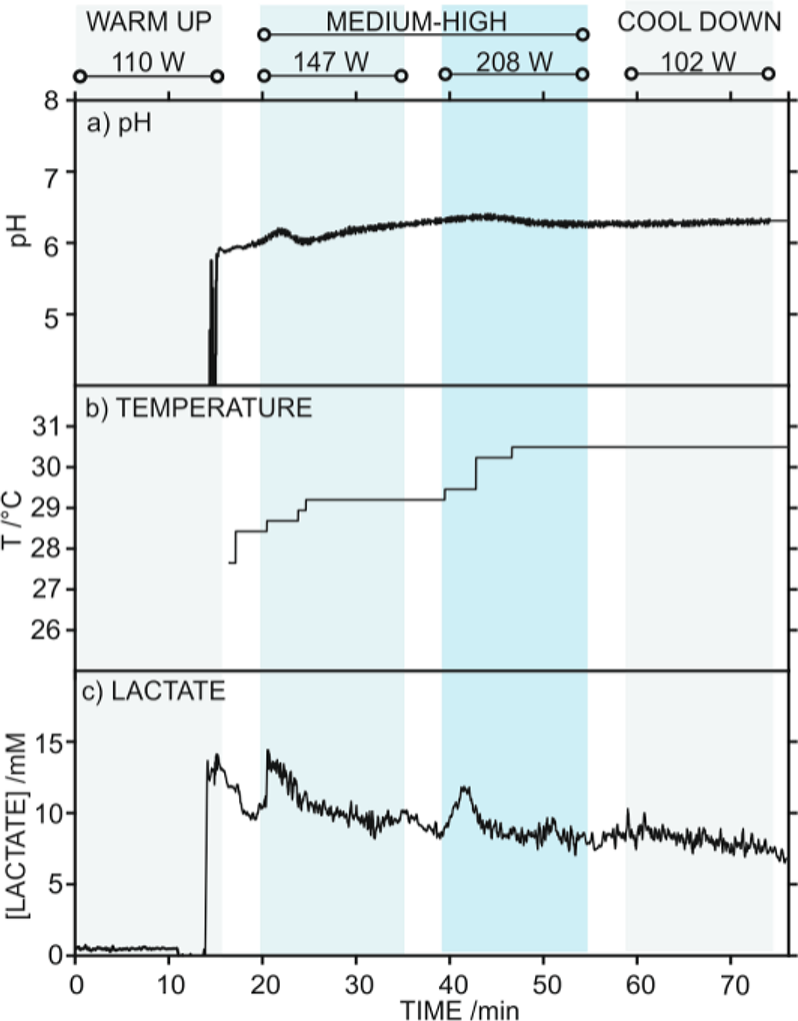
On-body test (T1) with the epidermal patch positioned in the back of the subject during stationary cycling exercise. Dynamic profiles for (a) pH, (b) temperature, and (c) sweat lactate. The workout power selected in each exercise period is provided.

Figure 3 shows the dynamic traces for pH, temperature, and lactate concentration obtained in a cycling test (T1) with the sensor attached to the back of the individual. The three-channel design of the microfluidic patch (Figure 1c) allowed sweat to reach all the sensors simultaneously through the inlets, which resulted in a meaningful signal from ca. 14 min of the test after perspiration is sufficient to reach the electrode surfaces. For example, this is manifested in a significant jump of the amperometric signal in the lactate biosensor (Figure 3c). This initial delay in registering the signal has been widely reported for wearable sensors attending to the need of the subject to reach the adequate perspiration rate during the warm-up activity, ranging from 8 to 19 min.^13,14,16^

Also, in the T1 on-body test, the pH was found to increase from ca. 5.8 to 6.4 as the level of intensity in the cycling activity was incremented (from 16 to 44 min), and then it decreased down to ca. 6.2 during the cool-down step (Figure 3a). Sweat temperature slowly increased during the exercise until a constant value of 30.5 °C was reached at 46 min (Figure 3b). Regarding the lactate levels (Figure 3c), a relatively high concentration was detected in the first sweat that reached the biosensor (ca. 13 mM at 14.5 min). Later, two increases in lactate concentration (13.5 and 11.7 mM) were observed at 20.5 and 41.5 min, corresponding to the initiation of low/middle and middle/high effort levels after the 5 min of rest in each case. After those increases, the lactate level relaxed down to ca. 9.6 and 8.4 mM in average.

Finally, the lactate concentration slowly decreases during the cool-down period. Overall, the observed increases in the lactate concentration as intensity workout was incremented may be related to lactate production during fast anaerobic metabolism, which is known to be reflected in increasing blood lactate levels and decreasing pH.^5^ However, it is not clear yet how this lactate production is reflected in the sweat lactate levels or how pH would be affected. Controversial results in this regard have been reported in the literature up to now.^6^ Advantageously, the further use of the new lactate biosensor in a massive study involving the simultaneous scrutiny of different parts of the body that provide lactate in passive *versus* active muscle, together with lactate blood correlations, will help to dissipating such physiological questions.

**Figure 4:**
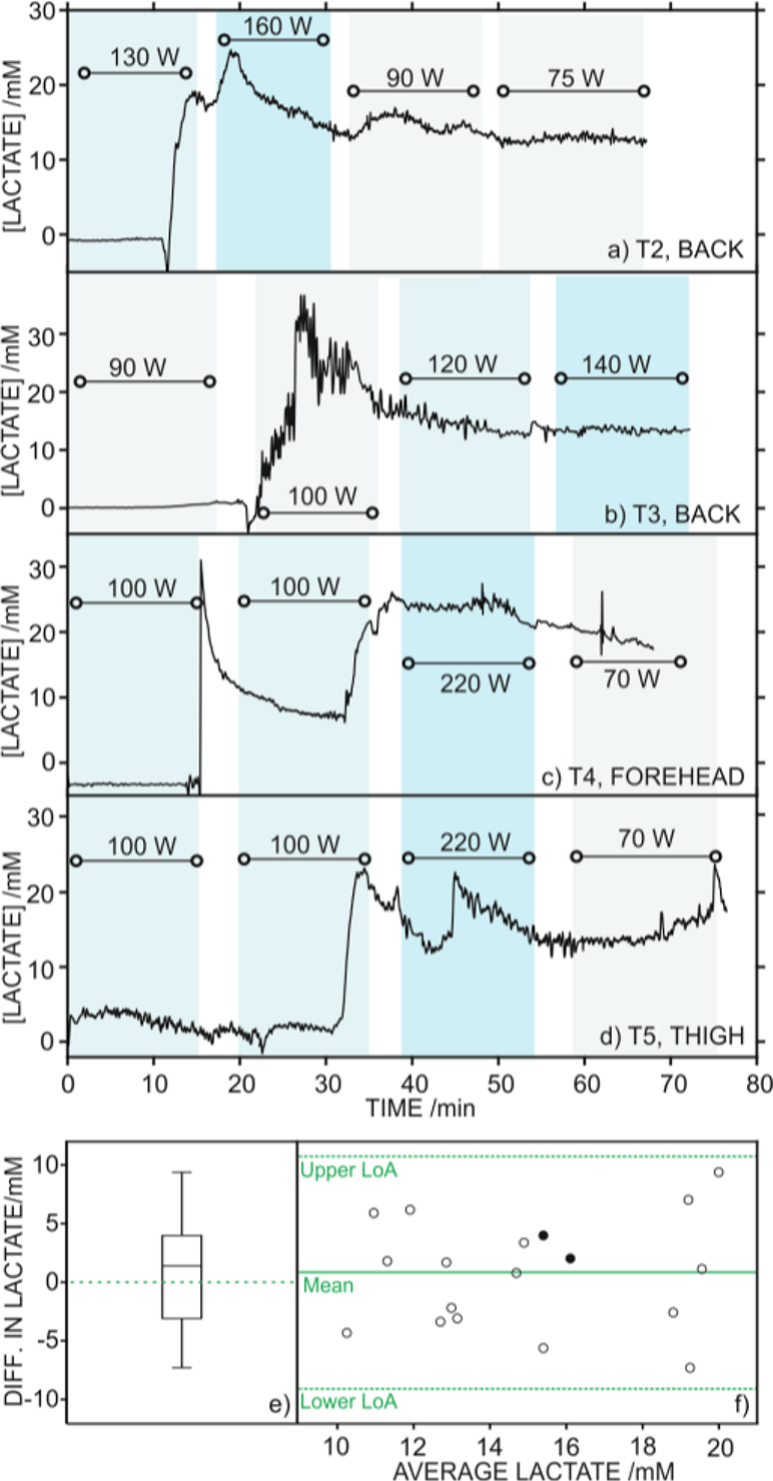
On-body tests (T2–T5) measuring sweat lactate during stationary cycling exercise with the epidermal patch positioned in the back (a and b), forehead (c), and thigh (d) of the individual. The workout power selected in each exercise period is provided. (e) Paired sample *t* test boxplot representing statistically analyzed differences in lactate concentration measured in on-body tests with the epidermal patch and by IC after sweat collection. (f) Bland–Altman plot showing the difference between both measurements against the average lactate concentration found in each sample. Full (●) and empty (○) circles represent results derived from iontophoresis and cycling, respectively. Orange horizontal lines represent average (solid) as well as lower and upper limits of agreement (LoA, dashed) calculated for a 95% of confidence level.

While the T1 test confirmed the nice applicability of the lactate, pH, and T sensors for on-body measurements through the three-channel epidermal patch, Figure 4a–d displays the other four on-body tests (T2–T5) in which only the lactate biosensor was used for validation purposes. In T2–T5, a microfluidic patch based on a single channel for the lactate biosensor was used (see Figure S2). Notably, special emphasis was provided to the validation of the lactate biosensor in these tests because the pH and T sensors have been widely validated in a recent paper by our group.^27^ A similar trend as that described for T1 was observed in T2–T5: first, sweat presented a high lactate concentration (ca. 22.7–32.0 mM) that slowly decreased afterward, with further increases in lactate associated to an increment in the power in the different cycling stages. Importantly, each subject required a different time to reach the adequate perspiration rate (i.e., from 12 to 31 min), which may be influenced by the physical fitness of each individual, the body location of the epidermal patch, and the selected power range.

In parallel to the T1–T5 on-body tests, sweat samples were collected every 15 min and analyzed with the IC. The average lactate concentration provided by the on-body measurements in the 15 min over sample collection was compared with the punctual values in the samples observed in the IC (Table S2 in the Supporting Information) by means of statistical analysis. A two-tailed paired sample *t* test (also known as dependent sample *t* test), which evaluates whether the average difference between the two analytical techniques is zero, was performed. Considering a 95% of confidence level, the calculated *t*-score *t*_calc_ = 0.74 was lower than the critical value *t*_stat_ = 2.11, meaning that there were no statistically meaningful differences between both (the biosensor and the IC) techniques. These results are graphically presented in Figure 4e, which displays a boxplot of the differences in lactate concentration provided by the sensor and IC. Most of the samples presented an absolute variation close to zero, with the first and third quartiles located at −3.2 and 4.0 mM, respectively.

A closer inspection to the individual agreement between samples can be observed through the Bland–Altman plot (Figure 4f), which represents the difference between both analytical techniques *versus* their average values. This type of plot allows one to identify trends and inconsistencies in variability across the considered lactate concentration range. In our case, the error distribution was homogeneous along the entire concentration range, meaning that discrepancy and variability are not dependent on the measured concentration. Nevertheless, the lower and upper limits of agreement (LoA) defined by a 95% of confidence level were rather wide, which may be attributed to the different time frequency in the measurements provided by each method.

The suitability of the developed lactate biosensor for healthcare purposes was additionally evaluated. Two healthy volunteers participated in this study (T6 and T7). The iontophoresis device (Macroduct) was first applied in the lower arm of the individual and then removed. Subsequently, the epidermal patch was attached in the same position where the anode of the iontophoresis device was placed (see Figure 1g). Simultaneously, sweat collection was carried out using a commercial elliptical sweat collector (Macroduct) after iontophoresis in the other arm. Lactate concentration was calculated as an average value of the biosensor signal recorded over a period of 15–30 min, depending on the time needed by each individual to generate an adequate volume of sweat.

Table S2 in the Supporting information shows the results obtained with the biosensor and the IC. Interestingly, very similar lactate concentration values were observed for both subjects (i.e., 15.1 and 13.4 mM), and variability against IC values was similar to that previously observed in on-body cycling tests (see Figure 4f, black circles). Overall, these results demonstrate that the developed wearable lactate biosensor is compatible with measurements *via* sweat induction by iontophoresis, which opens up the possibility of full integration of the two steps (i.e., iontophoresis and lactate detection) in a single device for further implementation in the healthcare field.

## Conclusions

We demonstrated a new strategy for lactate biosensing that provides an extended linear range of response covering expected levels in sweat (but also other biofluids) while preserving the biosensor response toward pH and temperature changes. This is achieved owing to the incorporation of an outer polymeric layer that modulates the lactate flux reaching the enzyme (lactate oxidase), which is immobilized in a Nafion matrix as the core of the biosensing mechanism. The sensor showed an acceptable response time, appropriate repeatability, reproducibility and reversibility, good resiliency, excellent selectivity, and low drift. Also, when integrated into an epidermal patch, the lactate concentration provided by the biosensor presented an extraordinary correlation with the results observed with a standardized method in the analysis of 22 sweat samples collected from 6 different subjects performing cycling or running. The new epidermal patch containing the lactate biosensor, but also pH and temperature sensors, is suitable for on-body sweat lactate, pH, and temperature monitoring while doing sport but also after sweat stimulation via iontophoresis. The patch can be positioned in the forehead, thigh, back and/or any place in the body for continuous sweat characterization encompassing perspiration. Overall, the developed epidermal patch with the new lactate biosensing concept is a promising tool for sweat analysis in both the sports tech and healthcare domains.

## Supporting Information Available

Sensor preparation, analytical assessment, and off-body validations (PDF)

## References

[ref1] Parrilla M.; Cuartero M.; Crespo G. A. Wearable potentiometric ion sensors. TrAC, Trends Anal. Chem. 2019, 110, 303–320. 10.1016/j.trac.2018.11.024.

[ref2] San-Millán I. Blood Biomarkers in Sports Medicine and Performance and the Future of Metabolomics. In High-Throughput Metabolomics; Springer: 2019; pp. 431–446. 10.1007/978-1-4939-9236-2_26 31119678

[ref3] Roda A.; Guardigli M.; Calabria D.; Calabretta M. M.; Cevenini L.; Michelini E. A 3D-printed device for a smartphone-based chemiluminescence biosensor for lactate in oral fluid and sweat. Analyst 2014, 139, 6494–6501. 10.1039/C4AN01612B. 25343380

[ref4] Sakharov D. A.; Shkurnikov M. U.; Vagin M. Y.; Yashina E. I.; Karyakin A. A.; Tonevitsky A. G. Relationship between lactate concentrations in active muscle sweat and whole blood. Bull. Exp. Biol. Med. 2010, 150, 83–85. 10.1007/s10517-010-1075-0. 21161059

[ref5] Karpova E. V.; Laptev A. I.; Andreev E. A.; Karyakina E. E.; Karyakin A. A. Relationship between sweat and blood lactate levels during exhaustive physical exercise. ChemElectroChem 2020, 7, 191–194. 10.1002/celc.201901703.

[ref6] Derbyshire P. J.; Barr H.; Davis F.; Higson S. P. J. Lactate in human sweat: a critical review of research to the present day. J. Physiol. Sci. 2012, 62, 429–440. 10.1007/s12576-012-0213-z. 22678934 PMC10717375

[ref7] Cuartero M.; Parrilla M.; Crespo G. Wearable potentiometric sensors for medical applications. Sensors 2019, 19, 363 10.3390/s19020363. PMC6359219 30658434

[ref8] Windmiller J. R.; Wang J. Wearable electrochemical sensors and biosensors: a review. Electroanalysis 2013, 25, 29–46. 10.1002/elan.201200349.

[ref9] Xuan X.; Kim J. Y.; Hui X.; Das P. S.; Yoon H. S.; Park J.-Y. A highly stretchable and conductive 3D porous graphene metal nanocomposite based electrochemical-physiological hybrid biosensor. Biosens. Bioelectron. 2018, 120, 160–167. 10.1016/j.bios.2018.07.071. 30173012

[ref10] Zaryanov N. V.; Nikitina V. N.; Karpova E. V.; Karyakina E. E.; Karyakin A. A. Nonenzymatic sensor for lactate detection in human sweat. Anal. Chem. 2017, 89, 11198–11202. 10.1021/acs.analchem.7b03662. 29065687

[ref11] Zhang Q.; Jiang D.; Xu C.; Ge Y.; Liu X.; Wei Q.; Huang L.; Ren X.; Wang C.; Wang Y. Wearable electrochemical biosensor based on molecularly imprinted Ag nanowires for noninvasive monitoring lactate in human sweat. Sens. Actuators, B 2020, 320, 128325 10.1016/j.snb.2020.128325.

[ref12] Mengarda P.; Dias F. A. L.; Peixoto J. V. C.; Osiecki R.; Bergamini M. F.; Marcolino-Junior L. H. Determination of lactate levels in biological fluids using a disposable ion-selective potentiometric sensor based on polypyrrole films. Sens. Actuators, B 2019, 296, 126663 10.1016/j.snb.2019.126663.

[ref13] Wang R.; Zhai Q.; An T.; Gong S.; Cheng W. Stretchable gold fiber-based wearable textile electrochemical biosensor for lactate monitoring in sweat. Talanta 2021, 222, 121484 10.1016/j.talanta.2020.121484. 33167206

[ref14] Jia W.; Bandodkar A. J.; Valdés-Ramírez G.; Windmiller J. R.; Yang Z.; Ramírez J.; Chan G.; Wang J. Electrochemical tattoo biosensors for real-time noninvasive lactate monitoring in human perspiration. Anal. Chem. 2013, 85, 6553–6560. 10.1021/ac401573r. 23815621

[ref15] Payne M. E.; Zamarayeva A.; Pister V. I.; Yamamoto N. A.; Arias A. C. Printed, flexible lactate sensors: Design considerations before performing on-body measurements. Sci. Rep. 2019, 9, 1–10. 31548553 10.1038/s41598-019-49689-7 PMC6757068

[ref16] Vinoth R.; Nakagawa T.; Mathiyarasu J.; Mohan A. M. V. Fully printed wearable microfluidic devices for high-throughput sweat sampling and multiplexed electrochemical analysis. ACS Sensors 2021, 6, 1174–1186. 10.1021/acssensors.0c02446. 33517662

[ref17] Lin K.-C.; Muthukumar S.; Prasad S. Flex-GO (Flexible graphene oxide) sensor for electrochemical monitoring lactate in low-volume passive perspired human sweat. Talanta 2020, 214, 120810 10.1016/j.talanta.2020.120810. 32278429

[ref18] Baker L. B. Sweating rate and sweat sodium concentration in athletes: a review of methodology and intra/interindividual variability. Sports Medicine 2017, 47, 111–128. 10.1007/s40279-017-0691-5. 28332116 PMC5371639

[ref19] Currano L. J.; Sage F. C.; Hagedon M.; Hamilton L.; Patrone J.; Gerasopoulos K. Wearable sensor system for detection of lactate in sweat. Sci. Rep. 2018, 8, 15890 10.1038/s41598-018-33565-x. 30367078 PMC6203741

[ref20] Liu Y.; Pharr M.; Salvatore G. A. Lab-on-skin: a review of flexible and stretchable electronics for wearable health monitoring. ACS Nano 2017, 11, 9614–9635. 10.1021/acsnano.7b04898. 28901746

[ref21] Gao W.; Emaminejad S.; Nyein H. Y. Y.; Challa S.; Chen K.; Peck A.; Fahad H. M.; Ota H.; Shiraki H.; Kiriya D.; Lien D. H.; Brooks G. A.; Davis R. W.; Javey A. Fully integrated wearable sensor arrays for multiplexed in situ perspiration analysis. Nature 2016, 529, 509–514. 10.1038/nature16521. 26819044 PMC4996079

[ref22] Anastasova S.; Crewther B.; Bembnowicz P.; Curto V.; Ip H. M.; Rosa B.; Yang G. Z. A wearable multisensing patch for continuous sweat monitoring. Biosens. Bioelectron. 2017, 93, 139–145. 10.1016/j.bios.2016.09.038. 27743863

[ref23] Li X.; Zhao L.; Chen Z.; Lin Y.; Yu P.; Mao L. Continuous electrochemical monitoring of extracellular lactate production from neonatal rat cardiomyocytes following myocardial hypoxia. Anal. Chem. 2012, 84, 5285–5291. 10.1021/ac300354z. 22607532

[ref24] Pribil M. M.; Laptev G. U.; Karyakina E. E.; Karyakin A. A. Noninvasive hypoxia monitor based on gene-free engineering of lactate oxidase for analysis of undiluted sweat. Anal. Chem. 2014, 86, 5215–5219. 10.1021/ac501547u. 24837858

[ref25] Pfeiffer D.; Setz K.; Schulmeister T.; Scheller F. W.; Lück H. B.; Pfeiffer D. Development and characterization of an enzyme-based lactate probe for undiluted media. Biosens. Bioelectron. 1992, 7, 661–671. 10.1016/0956-5663(92)85024-5.

[ref26] Tur-García E. L.; Davis F.; Collyer S. D.; Holmes J. L.; Barr H.; Higson S. P. J. Novel flexible enzyme laminate-based sensor for analysis of lactate in sweat. Sens. Actuators, B 2017, 242, 502–510. 10.1016/j.snb.2016.11.040.

[ref27] Wiorek A.; Parrilla M.; Cuartero M.; Crespo G. A. Epidermal patch with glucose biosensor: pH and temperature correction toward more accurate sweat analysis during sport practice. Anal. Chem. 2020, 92, 10153–10161. 10.1021/acs.analchem.0c02211. 32588617 PMC7467422

[ref28] Ashley B. K.; Brown M. S.; Park Y.; Kuan S.; Koh A. Skin-inspired, open mesh electrochemical sensors for lactate and oxygen monitoring. Biosens. Bioelectron. 2019, 132, 343–351. 10.1016/j.bios.2019.02.041. 30897541

[ref29] Enderle B.; Moser I.; Kannan C.; Schwab K. O.; Urban G. Interstitial glucose and lactate levels are inversely correlated with the body mass index: Need for in vivo calibration of glucose sensor results with blood values in obese patients. J. Diabetes Sci. Technol. 2018, 12, 341–348. 10.1177/1932296817730377. 28931321 PMC5851218

[ref30] Scheijen J. L. J. M.; Hanssen N. M. J.; Van de Waarenburg M. P. H.; Jonkers D. M. A. E.; Stehouwer C. D. A.; Schalkwijk C. G. L (+) and D (−) lactate are increased in plasma and urine samples of type 2 diabetes as measured by a simultaneous quantification of L (+) and D (−) lactate by reversed-phase liquid chromatography tandem mass spectrometry. Exp. Diabetes Res. 2012, 2012, 1 10.1155/2012/234812. PMC3310144 22474418

[ref31] Tékus É.; Kaj M.; Szabó E.; Szénási N. L.; Kerepesi I.; Figler M.; Gábriel R.; Wilhelm M. Comparison of blood and saliva lactate level after maximum intensity exercise. Acta Biol. Hung. 2012, 63, 89–98. 10.1556/ABiol.63.2012.Suppl.1.9. 22453744

[ref32] Thomas N.; Lähdesmäki I.; Parviz B. A. A contact lens with an integrated lactate sensor. Sens. Actuators, B 2012, 162, 128–134. 10.1016/j.snb.2011.12.049.

[ref33] Hernández-Ibáñez N.; García-Cruz L.; Montiel V.; Foster C. W.; Banks C. E.; Iniesta J. Electrochemical lactate biosensor based upon chitosan/carbon nanotubes modified screen-printed graphite electrodes for the determination of lactate in embryonic cell cultures. Biosens. Bioelectron. 2016, 77, 1168–1174. 10.1016/j.bios.2015.11.005. 26579934

[ref34] Fan Q.; Shan D.; Xue H.; He Y.; Cosnier S. Amperometric phenol biosensor based on laponite clay-chitosan nanocomposite matrix. Biosens. Bioelectron. 2007, 22, 816–821. 10.1016/j.bios.2006.03.002. 16624546

[ref35] Cui X.; Li C. M.; Zang J.; Yu S. Highly sensitive lactate biosensor by engineering chitosan/PVI-Os/CNT/LOD network nanocomposite. Biosens. Bioelectron. 2007, 22, 3288–3292. 10.1016/j.bios.2007.03.004. 17408948

[ref36] Perdomo J.; Sundermeier C.; Hinkers H.; Morell O. M.; Seifert W.; Knoll M. Containment sensors for the determination of L-lactate and glucose. Biosens. Bioelectron. 1999, 14, 27–32. 10.1016/S0956-5663(98)00104-3. 10028646

[ref37] Moser I.; Jobst G.; Urban G. A. Biosensor arrays for simultaneous measurement of glucose, lactate, glutamate, and glutamine. Biosens. Bioelectron. 2002, 17, 297–302. 10.1016/S0956-5663(01)00298-6. 11849926

[ref38] Reddy S. M.; Vadgama P. M. Ion exchanger modified PVC membranes—selectivity studies and response amplification of oxalate and lactate enzyme electrodes. Biosens. Bioelectron. 1997, 12, 1003–1012. 10.1016/S0956-5663(97)00055-9. 9451790

[ref39] Kisiel A.; Kałuża D.; Paterczyk B.; Maksymiuk K.; Michalska A. Quantifying plasticizer leakage from ion-selective membranes–a nanosponge approach. Analyst 2020, 145, 2966–2974. 10.1039/C9AN02621E. 32115595

[ref40] Grygolowicz-Pawlak E.; Bakker E. Thin layer coulometry with ionophore based ion-selective membranes. Anal. Chem. 2010, 82, 4537–4542. 10.1021/ac100524z. 20429515

[ref41] Liang R.; Yin T.; Qin W. A simple approach for fabricating solid-contact ion-selective electrodes using nanomaterials as transducers. Anal. Chim. Acta 2015, 853, 291–296. 10.1016/j.aca.2014.10.033. 25467471

[ref42] Cánovas R.; Padrell Sánchez S.; Parrilla M.; Cuartero M.; Crespo G. A. Cytotoxicity study of ionophore-based membranes: Toward on-body and in vivo ion sensing. ACS Sens. 2019, 4, 2524–2535. 10.1021/acssensors.9b01322. 31448593

[ref43] Matsumoto M.; Takagi T.; Kondo K. Separation of lactic acid using polymeric membrane containing a mobile carrier. J. Ferment. Bioeng. 1998, 85, 483–487. 10.1016/S0922-338X(98)80066-4.

[ref44] Matsumoto M.; Murakami Y.; Minamidate Y.; Kondo K. Separation of lactic acid through polymer inclusion membranes containing ionic liquids. Sep. Sci. Technol. 2012, 47, 354–359. 10.1080/01496395.2011.620582.

[ref45] Kalinichev A. V.; Solovyeva E. V.; Ivanova A. R.; Khripoun G. A.; Mikhelson K. N. Non-constancy of the bulk resistance of ionophore-based Cd2+–selective electrode: A correlation with the water uptake by the electrode membrane. Electrochim. Acta 2020, 334, 135541 10.1016/j.electacta.2019.135541.

[ref46] Wiorek A.; Cuartero M.; De Marco R.; Crespo G. A. Polyaniline Films as Electrochemical-Proton Pump for Acidification of Thin Layer Samples. Anal. Chem. 2019, 91, 14951–14959. 10.1021/acs.analchem.9b03402. 31691565

[ref47] Smith C. J.; Havenith G. Body mapping of sweating patterns in male athletes in mild exercise-induced hyperthermia. Eur. J. Appl. Physiol. 2011, 111, 1391–1404. 10.1007/s00421-010-1744-8. 21153660

[ref48] Radoi A.; Moscone D.; Palleschi G. Sensing the lactic acid in probiotic yogurts using an L-lactate biosensor coupled with a microdialysis fiber inserted in a flow analysis system. Anal. Lett. 2010, 43, 1301–1309. 10.1080/00032710903518716.

[ref49] Pilas J.; Yazici Y.; Selmer T.; Keusgen M.; Schöning M. J. Optimization of an amperometric biosensor array for simultaneous measurement of ethanol, formate, d-and l-lactate. Electrochim. Acta 2017, 251, 256–262. 10.1016/j.electacta.2017.07.119.

[ref50] Kucherenko I. S.; Soldatkin O. O.; Topolnikova Y. V.; Dzyadevych S. V.; Soldatkin A. P. Novel multiplexed biosensor system for the determination of lactate and pyruvate in blood serum. Electroanalysis 2019, 31, 1608–1614. 10.1002/elan.201900229.

[ref51] Parrilla M.; Ortiz-Gómez I.; Cánovas R.; Salinas-Castillo A.; Cuartero M.; Crespo G. A. Wearable potentiometric ion patch for on-body electrolyte monitoring in sweat: Toward a validation strategy to ensure physiological relevance. Anal. Chem. 2019, 91, 8644–8651. 10.1021/acs.analchem.9b02126. 31194514

