# Supplementary material for: Lactate Biosensing for Reliable On-Body Sweat Analysis
Source: ACS Sens. 2021 Jul 6;6(7):2763–71. doi: 10.1021/acssensors.1c01009 (PMC8397467; doi:10.1021/acssensors.1c01009)
Supplement: Supplementary file 1 — se1c01009_si_001.pdf [file se1c01009_si_001.pdf]

Supporting Information for:

Lactate Biosensing for Reliable On-Body Sweat Analysis

Xing Xuan, Clara Perez-Rafols, Chen Chen, Maria Cuartero and Gaston A. Crespo*

Department of Chemistry, School of Engineering Sciences in Chemistry, Biotechnology and Health, KTH Royal Institute of Technology, Teknikringen 30, SE-100 44, Stockholm, Sweden

*Corresponding author: gacp@kth.se.

Table of Contents

EXPERIMENTAL SECTION **SI-3**

Reagents, materials and instrumentation **SI-3**

Sensor fabrication **SI-3**

On-body and off-body tests. **SI-4**

Tables **SI-6**

Table S1. Compositions of the outer membranes used in lactate biosensors **SI-6**

Table S2. Lactate concentration obtained during on-body tests using the epidermal patch and IC measurements **SI-7**

Figures **SI-8**

Figure S1. Schematic draw of pH, T, and lactate sensors layer **SI-8**

Figure S2. Schematic draw of one channel cell **SI-8**

Figure S3. Characterization of the lactate biosensor in batch mode **SI-9**

Figure S4. Time trace of the chronoamperometric response **SI-9**

Figure S5. Resiliency test **SI-10**

Figure S6. Calibration curve for the pH and T sensors **SI-10**

Figure S7. Influence of the flow rate and reversibility studies **SI-10**

Figure S8. (a) Potentiometric response of Carbon/CNTs/ETH500 membrane **SI-11**

Figure S9. Off-body validation **SI-11**

Reference **SI-12**

EXPERIMENTAL SECTION

Reagents, materials and instrumentation. Potassium hexacyanoferrate(III) (CAS-13746-66-2, >98% purity), iron (III) chloride (CAS-7705-08-0, >97% purity), sodium L-lactate (CAS-867-56-1), chitosan (CAS-9012-76-4), Nafion perfluorinated resin solution 5% (CAS-31175-20-9), bis(2-ethylhexyl)sebacate (DOS) (CAS-122-62-3, ≤97% purity), polyurethane Tecoflex SG80A (PU) (CAS- 68400-67-9), high molecular weight poly(vinyl chloride) (PVC), tetradodecylammonium tetrakis(4-chlorophenyl)borate (ETH 500), and tetrahydrofuran (THF) (CAS-109-99-9) were purchased from Sigma-Aldrich. Analytical grade chloride salts of ammonium (CAS-12125-02-9), magnesium (CAS-7786-30-3), potassium (CAS-7447-40-7), sodium (CAS-7647-14-5), as well as sodium carbonate (CAS-497-19-8) and sodium phosphate (CAS-7558-79-4) were purchased from Sigma-Aldrich. Lactate oxidase (LOx) was purchased from Sorachim SA, Switzerland (LAX-18C). Silver/silver chloride (Ag/AgCl) ink and carbon ink were purchased from Henkel, German. The substrate material polyester sheet was purchased from RS components, Sweden. PU filament for the 3D printing of the sampling cell, TPU 95A, Ultimaker Material 1756 was purchased from Ultimaker B.V., Netherlands. All solutions were prepared in 18.2 MΩ cm^{-1} doubly deionized water (Milli-Q water systems, Merck Millipore). Macroduct for iontophoresis method was purchased from ELITechGroup, Netherlands. Artificial sweat was prepared as background solution for all the experiments, which contains 60 mM NaCl, 6 mM KCl, 5 mM NH_4Cl, 0.08 mM $MgCl_2$, 2.6 mM $NaHCO_3$ and 0.04 mM Na_2HPO_4 (pH 8.5)[1].

Amperometric measurements were performed with a potentiostat (Autolab, Metrohm Nordics AB, Sweden). For fundamental studies, a commercial single junction Ag/AgCl/3M KCl reference electrode (Metrohm Nordic, Sweden) and a platinum counter electrode (Metrohm Nordic, Sweden) were employed. For on-body and off-body validation, lactate content in sweat samples was additionally measured by ion chromatography (IC) (850 Professional IC, Metrohm AB, Sweden). The masks for screen printed electrode and adhesive transfer tape were designed with Auto-Cad software and fabricated using Silhouette Cameo cutter (Silhouette Inc., USA). The microfluidic cell was designed with Auto-Cad and printed with Ultimaker 3 printer (Ultimaker B.V., Netherlands). In the flow mode experiments, the samples flow through plastic tube (51 µm, SC0005T, ISMATEC, German) to the microfluidic cell by using peristaltic pump (ISMATEC IPC series, Cole-Parmer GmbH, Germany). Thickness measurements were carried out with a Dektak 150 Stylus surface profiler (Veeco, USA).

Sensor fabrication. For manual screen-printing of carbon and Ag/AgCl electrodes flexible substrate material polyester sheets were first cleaned with ethanol and dried with nitrogen for further use. Subsequently, the conductive inks were screen-printed on the polyester substrate and dried in the oven at 100 °C for 1 hour.

The modification to obtain the <u>lactate working electrode</u> consisted of a layer-by-layer deposition method. For the Prussian Blue (PB) layer, 3 µL of 0.1 M potassium ferricyanide and 3 µL 0.1 M iron (III) chloride were drop-casted on the carbon electrode, thoughtfully mixed, and left to react for 20 min at room temperature. Afterwards, the excess of PB was cleaned with 0.01 M HCl and the resulting layer was annealed in the oven at 100 °C for 1 hour. The deposition of the enzyme layer was carried out by drop-casting 2.5 µL of a solution containing 15 mg/mL LOx, 5 mg/mL BSA and 0.5%wt Nafion. This layer was

allowed to dry for 20 min at room temperature prior to depositing the outer polymeric membrane. For this purpose, a volume of 1.5 µL of a solution containing 3 mg/mL ETH 500, 33 mg/mL PVC and 66 mg/mL DOS in THF was drop-casted and dried at room temperature for 20 min. Finally, the electrode was conditioned overnight at 4 ºC in artificial sweat before further measurement. The same conditions were employed in the storing of the sensors.

A PANI-based pH sensor was used to detect pH in sweat. To deposit PANI on the carbon electrode, cycling voltammetry (from -0.2 to 0.8 V, 50 mV/s, 15 scans) was carried out in a solution containing 0.1 M aniline in 0.5 M H_2SO_4 and 0.1 M $NaPF_6$.[2] For the reference electrode, a Ag/AgCl screen-printed electrode was covered with a reference membrane containing 78 mg polyvinyl butyral and 50 mg NaCl mg in 1 mL methanol, as previously reported.[1] Electrodes were conditioned overnight in 3 M KCl. For the T sensor, a layer of 0.5 µL MWCNTs (1 mg/mL CNTs in THF) was drop casted between two carbon electrodes. Subsequently, the electrodes were dried in the oven for 1 hour at 100 °C to remove any water content.[1]

Each set of electrodes were connected to aluminum wires by using commercial 3-pin (lactate) or 2-pin (pH and temperature) connectors (5-520315-3-ND and A124763-ND, respectively, Digi-Key).

On-body and off-body tests. Before each on-body test, the lactate biosensors were calibrated in flow mode using solutions of 5, 10, 15, and 20 mM lactate in artificial sweat. Afterwards, the patch was attached to the skin of the volunteer by means of adhesive tape, and further secured with straps. Four different body locations were considered for the sensor placement (i.e., forehead, back, thigh, and arm) to test the sensor performance under different conditions: active/passive muscle, low/high sweating rate.

In cycling on-body tests, sweat samples were collected for validation purposes following a previously reported protocol.[3] Briefly, cotton pads are attached to the skin next to the sensor and sealed with Hydrofilm water-resistant tape to avoid evaporation and/or contamination. After a certain amount of time, the pad is removed and immediately squeezed using a syringe to extract the sweat. Sweat samples were stored in the fridge until further analysis.

Iontophoresis tests were performed using a commercially available medical grade device (Macroduct) according to the specified instructions. For each subject, iontophoresis was applied twice, one for on-body measurement with the epidermal patch and the other (in the other arm) for sweat collection. In the on-body measurements, the epidermal patch was attached to the same region right after the iontophoresis process. Sweat collection was performed with an elliptic collector for 15-40 min depending on the sweat rate of each specific subject.

In the off-body validation of the lactate biosensor, the collected sweat samples were analyzed in flow mode using the developed microfluidic cell but also with the IC (850 Professional IC, Metrhom, Switzerland) with conductivity detection and using a MetrosepA Supp 5 - 150/4.0 (ref. 6.1006.520) column, 1 mM $NaHCO_3$ / 3.2 mM Na_2CO_3 buffer with a flow rate of 0.8 mL min^{-1} as mobile phase and 100 mM H_2SO_4 as suppressor.

The on-body evaluation of the wearable device developed in the present work was performed in compliance with European and Swedish legislations and fundamental ethical principles, including those

reflected in the Chapter of Fundamental Rights in the European Union and the European Convention on Human Rights and its Supplementary Protocols (Dnr 2020-04206, Analys av jon- och biomolekylkoncentrationer i svett via sensorer applicerade på huden för att bestämma individens fysiologiska belastning).[4, 5] Furthermore, an informed consent was agreed with each subject before the on-body tests. This informed consent describes the aims, methods and implications of the research, the nature of the investigations and any benefits, risks or discomfort that may ensue during the test.

Tables

Table S1. Compositions of the outer membranes used in lactate biosensors.

Mmbrane	Solvent	PVC ($mg\ mL^{-1}$)	DOS ($mg\ mL^{-1}$)	TDMAC ($mg\ mL^{-1}$)	ETH 500 ($mg\ mL^{-1}$)
**M1**	THF	33	66	0	0
**M2**	THF	33	66	0.5	0
**M3**	THF	33	66	0	3
**M4**	THF	33	66	0	9

Table S2. Lactate concentration obtained during on-body tests using the epidermal patch and IC measurements. The difference between both methods are also provided in the table. Notably, the values observed with the lactate biosensor were calculated as the average of lactate concentration coinciding with the sweat collection time.

TEST	Subject	Average power (W)	Collection time (min)	Zone	Lactate (mM)	
					Sensor	IC
On-T1	1	110	0-15	Back	12.0	13.7
On-T1	1	147	15-30	Back	10.4	12.2
On-T1	1	208	30-45	Back	8.8	15.0
On-T1	1	102	45-60	Back	8.0	13.9
On-T2	2	130	0-15	Back	18.0	N/A
On-T2	2	160	15-30	Back	14.3	15.1
On-T2	2	90	30-45	Back	14.7	11.6
On-T2	2	75	45-60	Back	12.4	8.1
On-T3	2	90	0-15	Back	6.0	N/A
On-T3	2	100	15-30	Back	20.1	17.5
On-T3	2	120	30-45	Back	14.1	11.9
On-T3	2	140	45-60	Back	14.4	11.0
On-T4	3	100	0-15	Forehead	19.0	20.1
On-T4	3	100	15-30	Forehead	13.2	16.6
On-T4	3	220	30-45	Forehead	22.9	15.6
On-T4	3	70	45-60	Forehead	18.2	12.6
On-T5	3	145	30-60	Thigh	15.7	22.7
On-T5	3	122	0-60	Thigh	15.3	24.7
On-T6	4	-	30	Arm	15.1	17.1
On-T7	5	-	15	Arm	13.4	17.4

Figures

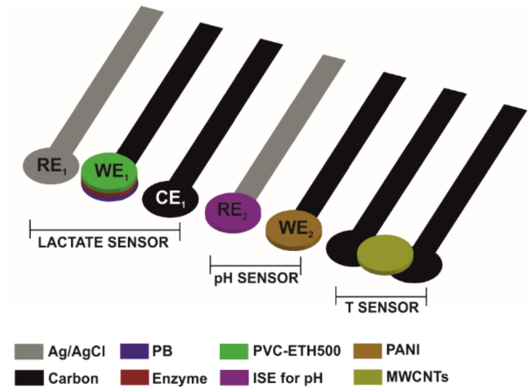

Figure S1. Schematic draw of pH, T, and lactate sensors layer.

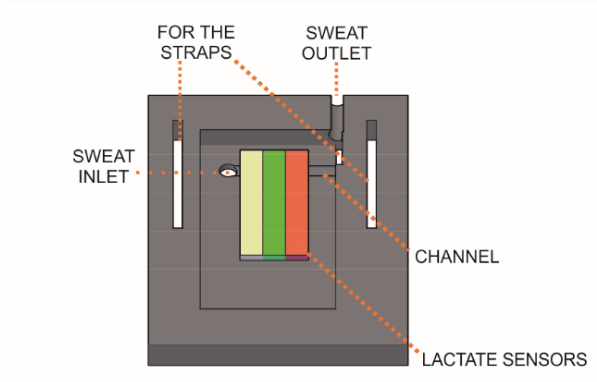

Figure S2. Schematic draw of one channel cell.

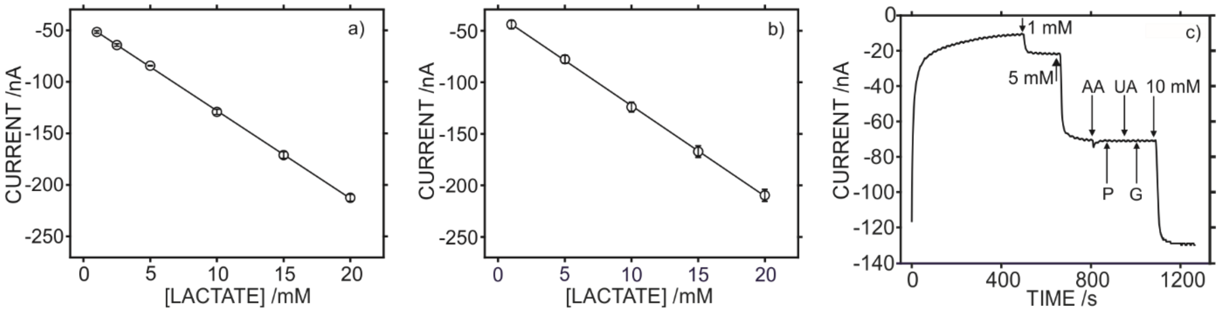

Figure S3. Characterization of the lactate biosensor in batch mode (stirred conditions, 300 rpm) in artificial sweat, with an applied potential of -0.05 V and using the screen-printed Ag/AgCl as the reference electrode. Average calibration graph with error bars corresponding to **(a)** three successive calibrations, and **(b)** three twin lactate sensors. **(c)** Response of lactate sensor to several interferences. The order of additions are: 1 mM lactate, 5 mM lactate, 0.1 mM ascorbic acid (AA), 0.1 mM uric acid (UA), 0.1 mM pyruvate (P), 0.25 mM glucose (G), and 5 mM lactate.

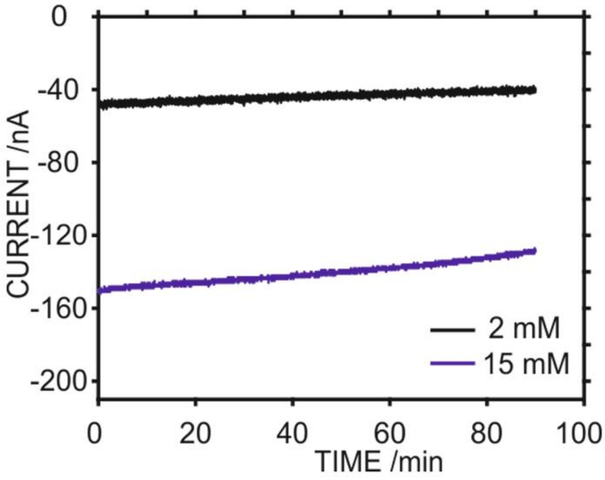

Figure S4. Time trace of the chronoamperometric response (<1 h) of the lactate biosensor in artificial sweat containing 2 mM (black) and 15 mM (purple) of lactate.

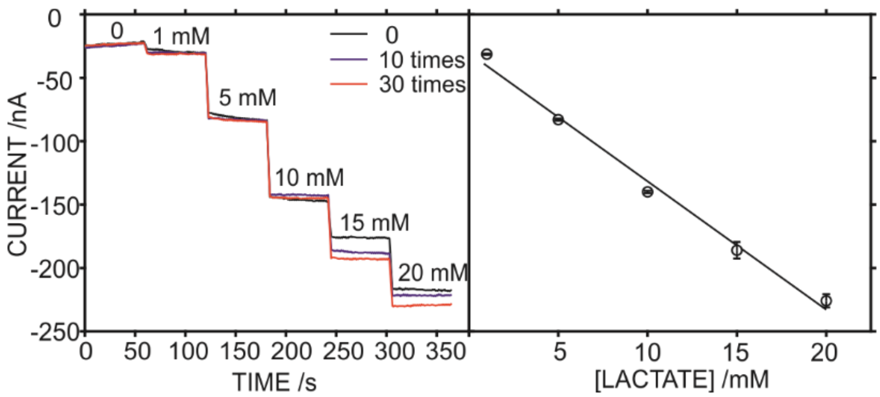

Figure S5. Resiliency test consisting of the application of an increasing number of torsion strain. Left: Dynamic responses for increasing concentrations of lactate. Right: corresponding calibration curves.

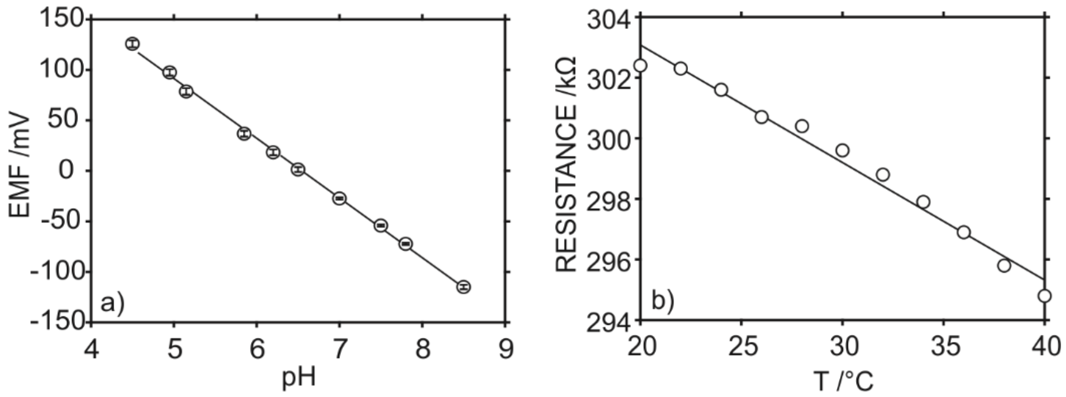

Figure S6. Calibration curve for the pH and T sensors.

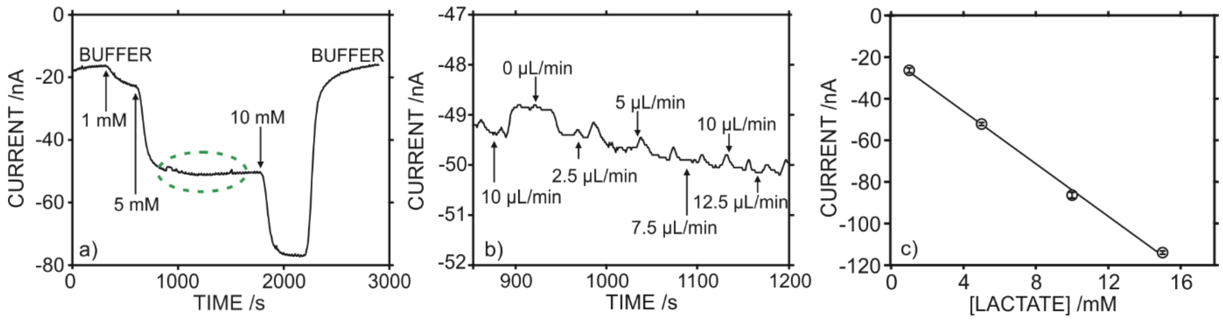

Figure S7. (a) Influence of the flow rate on the chronoamperometric response of the lactate biosensor tested in flow mode. **(b)** Zoom-in from the previous figure. **(c)** Average calibration curve with error bars for reversibility stud-ies.

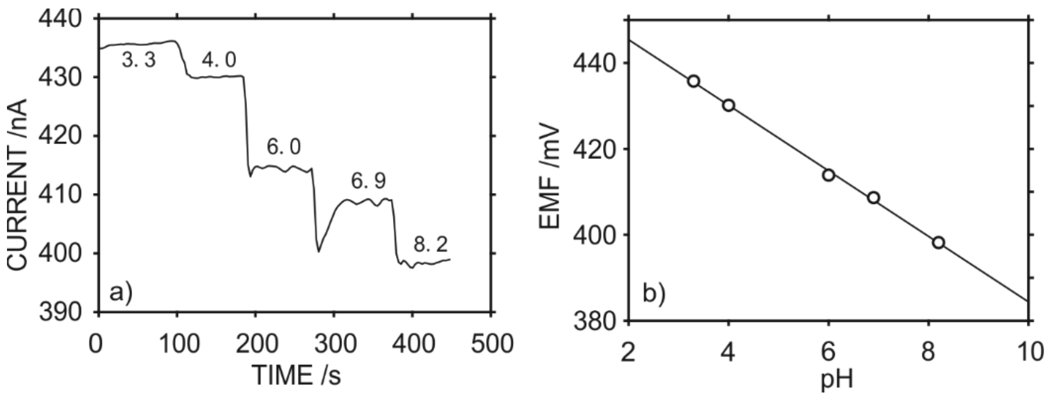

Figure S8. (a) Potentiometric response of an electrode composed of Carbon/CNTs/ETH500 membrane at different pH values. (b) Corresponding calibration graph.

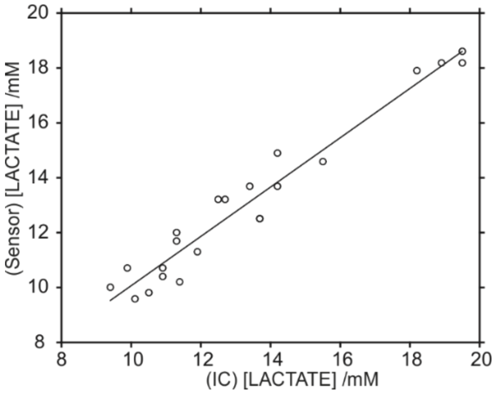

Figure S9. Correlation plot between lactate measurements in sweat performed off-body with the biosensor and with ion chromatography.

References

1. Wiorek, A.; Parrilla, M.; Cuartero, M.; Crespo, G. n. A., Epidermal Patch with Glucose Biosensor: pH and Temperature Correction toward More Accurate Sweat Analysis during Sport Practice. *Analytical Chemistry* **2020,** *92* (14), 10153-10161.

2. Wiorek, A.; Cuartero, M.; De Marco, R.; Crespo, G. A., Polyaniline Films as Electrochemical-Proton Pump for Acidification of Thin Layer Samples. *Analytical Chemistry* **2019,** *91* (23), 14951-14959.

3. Parrilla, M.; Ortiz-Gomez, I.; Canovas, R.; Salinas-Castillo, A.; Cuartero, M.; Crespo, G. A., Wearable potentiometric ion patch for on-body electrolyte monitoring in sweat: toward a validation strategy to ensure physiological relevance. *Analytical chemistry* **2019,** *91* (13), 8644-8651.

4. Convention, E., Charter of Fundamental Rights of the European Union. 2000.

5. Convention, E., European Convention on Human Rights. 1953.
